# Hybridization and massive mtDNA unidirectional introgression between the closely related Neotropical toads *Rhinella marina *and *R. schneideri *inferred from mtDNA and nuclear markers

**DOI:** 10.1186/1471-2148-11-264

**Published:** 2011-09-22

**Authors:** Fernando Sequeira, Davidson Sodré, Nuno Ferrand, José AR Bernardi, Iracilda Sampaio, Horacio Schneider, Marcelo Vallinoto

**Affiliations:** 1 CIBIO, Centro de Investigação em Biodiversidade e Recursos Genéticos, Campus Agrário de Vairão, Universidade do Porto, 4485-661 Vairão, Portugal; 2 Universidade Federal do Pará, Campus de Bragança, Alameda Leandro Ribeiro s/n 68.600-000, Bragança, Pará, Brazil; 3 Departamento de Biologia, Faculdade de Ciências da Universidade do Porto, Rua do Campo Alegre s/n, 4099-002 Porto, Portugal; 4 Instituto Federal de Educação, Ciência e Tecnologia, IFPA- Campus de Bragança, Travessa Santos Dumont, s/n 68.600-000, Bragança, Pará, Brazil

## Abstract

**Background:**

The classical perspective that interspecific hybridization in animals is rare has been changing due to a growing list of empirical examples showing the occurrence of gene flow between closely related species. Using sequence data from *cyt b *mitochondrial gene and three intron nuclear genes (*RPL9*, *c-myc*, and *RPL3*) we investigated patterns of nucleotide polymorphism and divergence between two closely related toad species *R. marina *and *R. schneideri*. By comparing levels of differentiation at nuclear and mtDNA levels we were able to describe patterns of introgression and infer the history of hybridization between these species.

**Results:**

All nuclear loci are essentially concordant in revealing two well differentiated groups of haplotypes, corresponding to the morphologically-defined species *R. marina *and *R*. *schneideri*. Mitochondrial DNA analysis also revealed two well-differentiated groups of haplotypes but, in stark contrast with the nuclear genealogies, all *R. schneideri *sequences are clustered with sequences of *R. marina *from the right Amazon bank (RAB), while *R. marina *sequences from the left Amazon bank (LAB) are monophyletic. An Isolation-with-Migration (IM) analysis using nuclear data showed that *R. marina *and *R. schneideri *diverged at ≈ 1.69 Myr (early Pleistocene), while *R. marina *populations from LAB and RAB diverged at ≈ 0.33 Myr (middle Pleistocene). This time of divergence is not consistent with the split between LAB and RAB populations obtained with mtDNA data (≈ 1.59 Myr), which is notably similar to the estimate obtained with nuclear genes between *R. marina *and *R. schneideri*. Coalescent simulations of mtDNA phylogeny under the speciation history inferred from nuclear genes rejected the hypothesis of incomplete lineage sorting to explain the conflicting signal between mtDNA and nuclear-based phylogenies.

**Conclusions:**

The cytonuclear discordance seems to reflect the occurrence of interspecific hybridization between these two closely related toad species. Overall, our results suggest a phenomenon of extensive mtDNA unidirectional introgression from the previously occurring *R. schneideri *into the invading *R. marina*. We hypothesize that climatic-induced range shifts during the Pleistocene/Holocene may have played an important role in the observed patterns of introgression.

## Background

Several recent studies have eroded the traditional perspective that interspecific hybridization is a rare phenomenon among animals, providing evidence that closely related taxa in different groups of organisms often share a history of introgressive hybridization (e.g. [1,2]). However, patterns of introgression appear heterogeneous across the genome, since the exchange of genomic regions between species depends greatly on their fitness effects or the fitness effects of linked regions [3]. In fact, there is now a growing list of studies showing extensive gene flow at some loci, whereas other loci remain differentiated, probably because some genomic regions associated with species-specific adaptations and/or reproductive isolation are less prone to introgress [4,5]. Furthermore, it is also possible that some alleles are more easily introgressed because they can either confer an advantage in an alternative environment or in a foreign genetic background [6]. The consequence of incomplete barriers to gene exchange is semipermeable or porous species boundaries [7,8].

While the recent availability of partially or fully sequenced genomes have greatly facilitated the study of patterns of introgression in model organisms, investigation of gene introgression in animals lacking those genomic resources is still a challenging task [9]. In fact, until recently, mtDNA has been the marker of choice for examining evolutionary relationships at the population level and among closely related species [10]. However, the study of a single gene is of particular concern because its genealogy may not truly reflect the history of populations or species, often leading to erroneous conclusions (e.g. [11]). Hence, taking advantage of recent advances in molecular methodologies such as the development of exon-primed intron-crossing (EPIC) primers (e.g. [12]), comparative studies of multiple gene genealogies, including nuclear and mtDNA markers, have been considered an alternative tool to examine the evolutionary history of diverging taxa and, in particular, for the detection of patterns of introgressive hybridization among closely related species of non-model organisms (e.g. [13,14]).

Both empirical and simulation studies have shown that interspecific hybridization can occur as the outcome of changing demographic conditions and/or species' distributional ranges [15,16]. In the northern hemisphere it is well-established that Pleistocene glacial cycles greatly affected the evolutionary history of many organisms, inducing population contractions and expansions following ecological changes (see [17], and references therein). Such processes often promoted contact between divergent evolutionary units or closely related taxa after climatic amelioration, leading to the occurrence of extensive hybridization and introgression (e.g [17,18]). In contrast, the consequences of Pleistocene climatic instability in the Neotropics since the early proposed "forest refugia" hypothesis (*sensu *[19]) are still a matter of intense debate. Whereas some recent studies support that hypothesis, suggesting that Pleistocene climatic fluctuations promoted extensive contraction and expansion of the forests following dryer and colder periods [20,21], others argue in favour of a permanent rain forest cover all over the Amazon basin during the last glacial period, even if the combination of reduced temperatures, precipitation and atmospheric CO2 concentrations have certainly produced changes in the composition and structure of forests [22]. Despite these differences, both scenarios suggest an important role of Pleistocene climatic oscillations as an engine of genetic structure modification for many Amazon species and communities.

**Figure 1:**
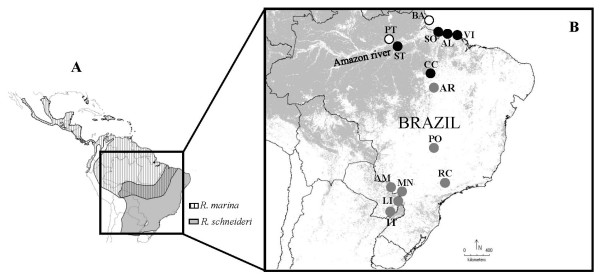
**Geographic distribution of *Rhinella marina *and *R. schneideri*, and sampling localities**. (A) Species distribution area [23]. (B) Sampling localities coded as in Additional file 1. White and black circles represent sample localities of *R. marina *from Left Amazon river bank (LAB) and Right Amazon river bank (RAB), respectively, and gray circles correspond to sample localities of *R. schneideri*. The map was designed using DIVA-GIS [96]. Gray layer is the land cover according to the tree cover, broadleaved, and evergreen option.

Two closely related anuran species widely distributed in South America (*Rhinella marina *and *R. schneideri*: Bufonidae) provide a compelling case study to investigate interspecific hybridization. Across South America, *R. marina *is restricted to rainforests of the Amazonian region, whereas *R. schneideri *is typically found in dry forests (Caatinga and Cerrado) along northeast and south of Brazil, including Paraguay, Bolivia, Uruguay and Argentina [23]. These species are morphologically similar (but diagnosable by the presence of tibial glands in *R. schneideri*) and have essentially a parapatric distribution, but may occur in sympatry along ecological transitions between humid and dry forests (Figure 1). Previous phylogenetic analyses of the *R. marina *group based on mtDNA [24,25] suggested a division in two major clades: one including *R. veredas*, *R. cerradensis*, *R. jimi*, *R. marina*, *R*. *schneideri*, and *R. poeppigii *(north-central clade), and another including *R. arenarum*, *R. rubescens*, *R. achavali*, and *R. icterica *(south-central clade). Interestingly, when only samples of *R. marina *located south of the Amazon river are used this species together with *R*. *schneideri*, *R. jimi *and *R. poeppigii *formed an unresolved polytomy [25]. While Vallinoto et al. [25] did not exclude that retention of ancestral polymorphism may explain this observation, they suggested instead that it could reflect past or current hybridization events. Considering the different habitat preferences of *R. marina *and *R. schneideri*, it is possible that strong environmental changes after the Last Glacial Maximum (LGM) in South America [26] may have impacted dramatically their demography and distribution ranges, with consequences on the evolutionary trajectories of both taxa. Finally, we note that *R. marina *is a highly invasive species whose history, ecology and demography have been studied in detail in Australia and in the Hawaian archipelago, but not in its native range [27-29].

In this study, we focused on *R. marina *populations from the eastern Amazonian region as well as on populations of *R. schneideri *representative of its distributional range. We used one mitochondrial gene and three nuclear introns to examine patterns of polymorphism and divergence within and between the two species, and addressed three major questions: i) are *R. marina *and *R. schneideri *well-differentiated at the nuclear level and how does this differentiation compares to the mtDNA level? ii) is there evidence of hybridization between the two species or do the results conform more with the retention of ancestral polymorphism? and iii) can we infer post-glacial expansion movements and is there evidence for the occurrence of a hybrid zone?

## Results

### Mitochondrial DNA analysis

**Figure 2:**
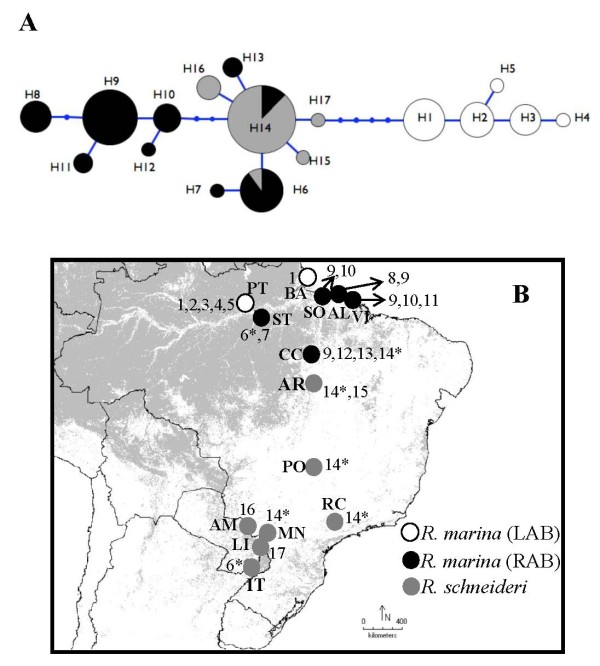
**Mitochondrial *cyt b *genealogy**. (A) Haplotype genealogy from Maximum likelihood of *cyt b *gene tree performed in the software Haploviewer. The model of nucleotide substitution for ML of *cyt b *gene was K81uf + G (0.047). (B) Geographic distribution of haplotypes present in *R. marina *from Left Amazon river bank (LAB) and Right Amazon river bank (RAB), and in *R. schneideri*. The Circle area of each haplotype, coded as a number (Additional file 1), is proportional to its frequency. Asterisks mark haplotypes present in both species. Dots represent inferred unsampled or extinct haplotypes.

A fragment of 327 base pairs (bp) of the *cyt b *gene was obtained from 92 individuals: 65 of *R. marina*, and 27 of *R. schneideri *(Table 1). The ingroup alignment revealed 17 distinct haplotypes, from which only five are present in *R. schneideri*. The genealogical relationships between the haplotypes (Figure 2A) showed two main haplotype groups, one corresponding to *R. marina *sequences from left Amazon bank (LAB), and the other comprising sequences of *R. marina *from the right Amazon bank (RAB) and from *R. schneideri*. Within the RAB group, individuals from the northwestern population (ST) and from all northeastern populations (SO, AL and VI) were included in two different sub-groups. Individuals from central-south Amazonian forest (CC) were clustered in both sub-groups of RAB (Figure 2A and Figure 2B). With the exception of Paraguay localities (IT, LI and AM), all sampled populations of *R. schneideri *exhibit haplotype H14, which was fixed in PO, RC and MN. This haplotype was also present in three *R. marina *individuals from Canaã dos Carajás (CC) (Figure 2A andAdditional file 1).

**Table 1:** Summary statistics, neutrality and recombination tests for each locus.

Loci/species	N^(1)^	Total Sites	H	S	*π*	*θ*	Tajima's *D*	Fu's *Fs*	R_2_	Rm	*Φw*
***RPL9***											
*R. marina*	64	504 (495)	19	22	0.752	0.939	-0.619	-5.138*	0.099	3	0.665
LAB	20	504 (497)	7	9	0.324	0.510	-1.256 (0.08)	-1.882	0.100	0	
RAB	44	504 (496)	15	22	0.917	1.019	-0.329	-2.892	0.083	2	
*R. schneideri*	30	504 (489)	11	16	0.715	0.877	-0.632	-2.470	0.104	3	0.322
***RPL3***											
*R. marina*	80	678 (662)	19	23	0.525	0.701	-0.760	-4.692 (0.07)	0.149	2	0.222
LAB	16	678 (665)	6	9	0.426	0.407	0.164	0.252	0.088	0	
RAB	64	678 (665)	14	17	0.456	0.540	-0.469	-2.415	0.074	2	
*R. schneideri*	30	678 (675)	10	7	0.328	0.261	0.759	-2.689 (0.07)	0.153	2	0.273
***C-myc***											
*R. marina*	52	585 (578)	10	8	0.144	0.340	-1.599*	-6.328*	0.190	0	1.0
LAB	10	585 (578)	3	2	0.113	0.120	-0.184	-0.272	0.055*	0	
RAB	42	585 (578)	10	8	0.149	0.357	-1.687*	-6.706**	0.053*	0	
*R. schneideri*	24	585 (577)	6	5	0.209	0.232	-0.279	-1.399	0.120	0	1.0
***Cyt b***											
*R. marina*											
LAB	22	327	5	4	0.338	0.336	0.017	-0.7394	0.146	-	-
RAB	43	327	9	10	0.904	0.707	0.819	0.01	0.140	-	-
*R. schneideri*	27	327	5	4	0.131	0.317	-1.555*	-3.074**	0.083	-	-

N, number of sequences; H, number of haplotypes; S, number of segregating sites; *π*, nucleotide diversity; *θ*, Watterson population mutation parameter [71]; Rm, minimum number of recombination events [73]; and, *Φw *statistic p-value [74].

* *P *< 0.05; ** *P *< 0.01. ^(1) ^Considering the two copies in the case of nuclear loci.

### Nuclear genealogical analysis

For the *RPL9 *nuclear fragment, we obtained 94 sequences from 47 individuals (32 *R*. *marina *and 15 *R. schneideri*). The ingroup alignment (504 bp) revealed 30 haplotypes (19 and 11 for *R. marina *and *R. schneideri*, respectively), defined by 41 polymorphic sites and 29 parsimoniously informative sites. While a minimum of three recombination events (Rm) was found in *RPL9 *for *R. marina *and *R. schneideri*, no statistical evidence for the occurrence of recombination was detected (*P *> 0.05) using the *Φw *test (Table 1 and Additional file 1).

For the *RPL3 *nuclear fragment, we obtained 110 sequences from 55 individuals (40 *R. marina *and 15 *R. schneideri*). The ingroup alignment (678 bp) revealed 29 haplotypes (19 and 10 for *R. marina *and *R. schneideri*, respectively) defined by 27 polymorphic sites, from which 23 were parsimoniously informative sites. A minimum number of two recombination events (Rm) was inferred for both species, but no statistical support was found when the *Φw *test (*P *> 0.05) was used (Table 1 and Additional file 1).

For the *c-myc *nuclear fragment, we obtained 76 sequences from 38 individuals (26 *R*. *marina *and 12 *R. schneideri*). The ingroup alignment (585 bp) revealed 16 haplotypes (10 and 6 for *R. marina *and *R. schneideri*, respectively) defined by 15 polymorphic sites, from which 13 were parsimoniously informative sites. According to both Rm and *Φw *tests, no evidence for recombination was observed in *c-myc *sequences (Table 1 and Additional file 1).

**Figure 3:**
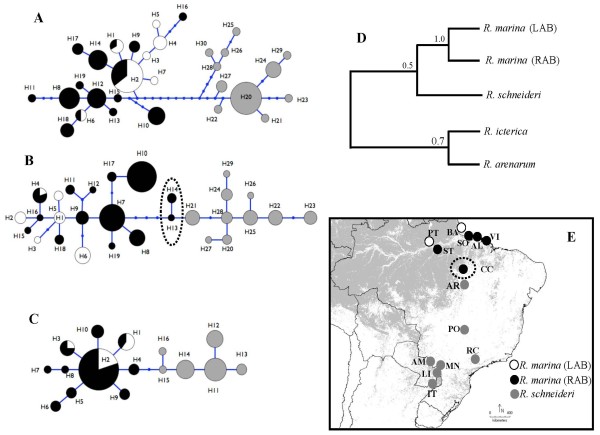
**Nuclear genealogies**. (A) Haplotype genealogy from Maximum likelihood analysis of *RPL9*, (B) *RPL3 *and (C) *c-myc *performed in the software Haploviewer. Models of nucleotide substitution used for ML were: TN93+G (0.095) for *RPL9*; TVM + G (0.014) for *RPL3*; and, K81uf + G (0.015) for *c-myc*. (D) Bayesian inference of species tree based on nuclear data performed in *BEAST. Bayesian posterior probabilities are above branches. (E) Geographic distribution of haplotypes observed in *R. marina *from Left Amazon river bank (LAB) and Right Amazon river bank (RAB), and in *R. schneideri*. The circle area of each haplotype, coded as a number (Additional file 1), is proportional to its frequency. Haplotypes delimited by a dotted line represent sequences clustered within *R. schneideri *that are present in three heterozygous *R. marina *individuals. Dots represent inferred unsampled or extinct haplotypes.

For all nuclear loci, haplotypes clustered in two groups corresponding to *R. marina *and *R. schneideri*, separated by a minimum of two (*c-myc*) and a maximum of twelve (*RPL9*) fixed differences (Figure 3A, Figure 3B and Figure 3C). The only exception to the species-specific group of haplotypes occurred in *RPL3*, where three individuals of *R. marina *from Canaã dos Carajás (CC) were heterozygous, and their haplotypes clustered both within *R. Schneideri *(H13 and H14) and the *R. marina *group (H7, H8 and H10) (Figure 3B and Figure 3E). Within the two geographically-defined groups of *R. marina*, we observed shared polymorphism (Table 2).

**Table 2:** Polymorphism and sequence divergence for each locus.

	Average Differences	D_xy _(%)	D_a _(%)	ψ	ψ 1	ψ 2	ψ Shared
***RPL9***							
LAB × RAB	3.843	0.776	0.206	0	1	13	8
LAB × *R. schneideri*	14.167	2.921	2.451	7	8	16	0
RAB × *R. schneideri*	15.081	3.115	2.288	6	20	16	1
							
***RPL3***							
LAB × RAB	4.436 (4.270)*	0.670 (0.642)*	0.226 (0.227)*	0	6	14 (9)*	3
LAB × *R. schneideri*	10.102	1.519	1.1139	6	8	6	1
RAB × *R. schneideri*	9.158 (9.402)*	1.377 (1.407)*	0.982 (1.040)*	1 (5)*	17 (12)*	7	0
							
***C-myc***							
LAB × RAB	0.81	0.138	0.006	0	0	7	2
LAB × *R. schneideri*	5.317	0.926	0.762	3	2	5	0
RAB × *R. schneideri*	5.270	0.918	0.736	2	8	4	1
							
***Cyt b***							
LAB × RAB	7.498	2.292	1.672	4	4	10	0
LAB × *R. schneideri*	7.029	2.149	1.915	4	4	4	0
RAB × *R. schneideri*	3.258	0.996	0.479	0	8	2	2

*D_xy_*, average pairwise number of nucleotide substitutions per site; *D_a_*, average pairwise net nucleotide divergence; ψ, number of polymorphic sites exclusive of each group or species; ψ 1; refers to the first species/group listed in each pairwise comparison.

*Removing the *R. schneideri *haplotypes (H13 and H14) present in three *R. marina *heterozygous from population CC (see Fig. 3).

The reconstruction of the phylogenetic species tree based on the three nuclear genes is shown in Figure 3D. While none of the three nuclear loci examined in this study was completely sorted with regard to LAB and RAB groups of *R. marina*, a Bayesian species tree inference method produced a high posterior probability (*P *= 1.0) for their sister-taxa relationship. The placement of *R. schneideri *with respect to *R. marina *(LAB)/*R. marina *(RAB) and the weakly supported clade *R. icterica/R. arenarum *were unresolved.

### Patterns of nucleotide variation and divergence

Patterns of nucleotide diversity within each species and for each main clade identified using the phylogenetic analysis are summarized in Table 1. Levels of diversity were highly variable across loci and within species. Across nuclear loci, *c-myc *exhibited lower levels of variability than *RPL3 *and *RPL9*. At the species level, *R. schneideri *presented, in general, lower levels of diversity when compared to *R. marina*, except for *c-myc*. One of the most remarkable differences was detected at the mtDNA *cyt b *gene, where *π *was considerably lower for *R*. *schneideri *(*π *= 0.131) than was observed in both *R. marina *groups (*π *= 0.338 and *π *= 0.904 for LAB and RAB, respectively). Tajima's *D*, Fu's *Fs *and R_2 _statistics were highly variable among loci, although essentially non-significant (P > 0.05). The main exceptions occurred in *R. schneideri *for the *cyt b *gene and in *R. marina *RAB group for the *c-myc *nuclear locus. In the former *cyt b *had a significantly negative skew for Tajima's *D *and Fu's *Fs *tests, while in the latter all tests showed significant deviations from neutral expectations for *c-myc*, also exhibiting negative values for Tajima's *D *and Fu's *Fs *tests, and a low value for R_2 _(Table 1).

At the mtDNA level, the divergence measured by *D_a _*and *D_xy _*between samples of *R*. *marina *from RAB and LAB was approximately twofold higher than the divergence between *R. marina *from RAB and *R. schneideri*. The average distance between the RAB group and *R. schneideri *ranged between 0.48% and 1.0% for *D_a _*and *D_xy_*, respectively. RAB and LAB groups showed values of divergence of 1.67% and 2.29% for *D_a _*and *D_xy_*, respectively (Table 2). At the nuclear level, the divergence between *R. marina *and *R. schneideri *ranged from a minimum of 0.74% and 0.93% (*c-myc*) to a maximum of 2.45% and 3.12% (*RPL9*) for *D_a _*and *D_xy_*, respectively.

### Divergence times and coalescent simulations

The approximate posterior density curves of the model parameters which resulted from the analyses using the Isolation-with-Migration model implemented in the software IMa2 are shown in Additional file 2. Posterior distributions of parameter estimates were consistent across replicate runs with effective sample sizes (ESSs) values>120, and there was no evidence of trends in the ASCII-based parameter trendline plots. However, the right tail of the posterior density curves was often relatively flat and failed to reach zero in the estimates of divergence time and ancestral effective population size. By consequence, in most of these cases the 95% HPD (highest posterior density) estimates were not reliable, reflecting some degree of uncertainty, and so they should be interpreted with caution (Table 3). Based on nuclear data, our IMa2 analysis showed that the split between *R. marina *and *R. schneideri *was estimated to be ≈ 1.69 Myr, and the split between *R. marina *populations from RAB and LAB occurred at ≈ 0.33 Myr. For the mtDNA analysis the split between LAB and RAB occurred at ≈ 1.59 Myr. We also estimated the posterior distributions of IMa2 parameters considering the two clades uncovered by mtDNA phylogeny (LAB and RAB + *R. schneideri*; Figure 2 and table 3). This analysis indicated that populations on the opposite banks of the Amazon River diverged at ≈ 1.67 Myr, which was very similar to the divergence time estimates inferred for *R. marina *and *R. schneideri *based on nuclear loci alone. Likewise, when we used all four loci simultaneously, assuming that mtDNA from the RAB group belongs to *R. schneideri*, IMa2 also resulted in estimates of time since divergence between *R*. *marina *and *R. schneideri *beginning in the early Pleistocene (≈ 1.73 Myr). To test whether estimated levels of the effective number of migrant gene copies per generation (i.e., the population migration rate; 2Nm) for each pairwise comparison were significantly different from zero we used the LLR tests implemented in IMa2. Our results indicated a significant level of gene flow only from *R. marina *LAB to RAB populations when information of nuclear genes is used (2Nm = 2.15; 95% HPD = 0.75 - 5.05; LLR = 8.71; *P *< 0.01).

**Table 3:** Maximum-likelihood estimates and 95% HPD (highest posterior density intervals) of divergence time (**t**) inferred by Isolation-with-Migration model (IMa2) for different pairwise comparisons.

Comparison	Markers	t (Myr.)
(LAB + RAB) × *R. schneideri *^a^	nDNA	1.690 (0.551-9.823)?
LAB × RAB ^b^	nDNA	0.325 (0.068-7.134)?
LAB × RAB ^c^	mtDNA	1.592 (0.861-6.444)
LAB × (RAB + *R. schneideri*) ^d^	mtDNA	1.668 (0.759-11.076)?
(LAB + RAB) × (RAB + *R. schneideri*) ^e^	nDNA + mtDNA	1.734 (0.644-8.936)?

^a ^*R. marina *(LAB + RAB) and *R. schneideri *using nuclear loci.

^b ^*R. marina *(LAB) × *R. marina *(RAB) using nuclear loci and mtDNA. ^c ^*R. marina *(LAB) × *R. marina *(RAB) using mtDNA.

^d ^*R. marina *(LAB) × [*R. marina *(RAB) + *R. schneideri*] using mtDNA.

^e ^*R. marina *and *R. schneideri *combining all markers and assuming that mtDNA from RAB population belongs to *R. schneideri*. ? values corresponding to the cases where parameters could not be reliably estimated.

Given that we found mtDNA shared variation between species, we conducted coalescent simulations to investigate whether the conflicting signal found in the mtDNA versus nuclear DNA-based phylogenies could be explained by incomplete lineage sorting. We simulated mtDNA sequence datasets under a model with no gene flow and using parameter estimates of divergence times and N_e _(effective population size) inferred from the IMa2 analysis using nuclear loci. The model included two historical events: i) an ancestral haploid population with Ne≈63,000 that splits into LAB (with Ne≈69,000) and RAB (with Ne≈190,000) populations at 325,000 generations in the past; and ii) an ancestral haploid population with approximately Ne≈103,000 that splits into two descendant populations with Ne≈269,000 (*R. marina*; RAB+LAB) and Ne≈143,000 (*R*. *schneideri*) at 1,690,000 generations ago. Under this scenario, the monophyly of *R. schneideri *was recovered in 99.7% of the simulated mtDNA gene trees, therefore rejecting the hypothesis of incomplete lineage sorting.

## Discussion

Our analysis of patterns of genetic variability and phylogenetic relationships in a comprehensive set of *R. marina *and *R. schneideri *populations from South America clearly suggests a compelling case of massive unidirectional mtDNA introgressive hybridization between these taxa. More specifically, we found that i) both species are highly divergent at the nuclear level, a fact that is not observed at the mtDNA level, ii) the previously described mtDNA/nuclear discordance in populations of *R. marina *are more easily explained by interspecific hybridization and not by the retention of ancestral polymorphism, as revealed by coalescent simulations of mtDNA phylogeny under the speciation history inferred from nuclear genes, and iii) the application of the Isolation-with-Migration model (IM) to nuclear data suggests that *R. marina *and *R. schneideri *diverged at about 1.7 Myr ago, while estimates of divergence between *R. marina *LAB and RAB populations suggest a split time in the middle Pleistocene (≈ 0.33 Myr). This time of divergence is not consistent with the split between the two main mtDNA clades LAB and RAB + *R. schneideri *(≈ 1.67 Myr). Below, we discuss all these issues in detail and suggest that the most likely explanation for the cytonuclear discordance resulted from the capture of *R. schneideri *mtDNA by the invading *R. marina*.

### Introgressive hybridization *vs *retention of ancestral polymorphism

Conflicting mitochondrial and nuclear gene trees among closely related species are often taken as an indication of mtDNA introgression due to interspecific hybridization (e.g [30]). However, the alternative scenario of incomplete lineage sorting may also result on the frequently observed pattern of mtDNA paraphyly or polyphyly. While the lower effective population size of mtDNA implicates that lineages sort out more easily among species than in the nuclear genome [1,31], the fact is that in most cases the two alternative hypotheses are not explicitly tested.

Our phylogenetic analysis of *cyt b *gene sequences showed that both taxa do not form monophyletic groups, revealing instead two major groups of haplotypes, one corresponding to *R. marina *samples from LAB, and the other including both *R. marina *from RAB and *R*. *schneideri *(Figure 2). This close relationship between *R. marina *and *R. schneideri *was already reported by Vallinoto et al. [25] based on a detailed phylogenetic analysis of the *R. marina *group using a larger mtDNA fragment (ca. 2000 bp). These results are in stark contrast with the phylogenetic pattern observed for nuclear loci both inferred by network haplotype genealogical relationships and by the species tree coalescent method that takes into account differential lineage sorting across markers (Figure 3). Networks of nuclear loci are basically concordant in revealing two highly divergent groups of haplotypes, corresponding to morphological-defined *R. marina *and *R. schneideri *species. The three nuclear loci exhibited a lack of shared haplotypes and a number of fixed differences between the two species that ranged from two to twelve, while a pattern of shared polymorphism was observed within the two geographically-defined groups of *R. marina *(LAB and RAB) (Figure 3; Table 2). The only exception to these species-specific groups of haplotypes occurred at the *RPL3 *gene, where three *R. marina *individuals sampled in Canaã dos Carajás (CC) shared haplotypes clustered within both *R. schneideri *and *R. marina *species. The Bayesian species tree reconstruction corroborates the monophyly of *R. marina*, supporting a close relationship between LAB and RAB populations (Figure 3D).

The close relationship between *R. marina *populations based on nuclear loci is supported by the use of a coalescent-based Isolation-with-Migration model (IMa2). This analysis suggested that *R. marina *and *R. schneideri *likely diverged at ≈ 1.7 Myr, while the inferred split time between *R. marina *LAB and RAB populations was at ≈ 0.33 Myr ago. In contrast, the divergence time for the different pairwise comparisons across the Amazon River bank populations based on mtDNA (including between LAB and RAB *R. marina *populations) or combining all loci (see Table 3 and Additional file 2) is always similar to that obtained for *R. marina *and *R. schneideri *(≈ 1.6 - 1.7 Myr). Coalescent simulations of mtDNA phylogeny under the speciation history inferred from nuclear genes clearly show that this conflicting signal found in the mtDNA-based phylogeny relative to the nuclear DNA could not be explained by incomplete lineage sorting. In fact, the monophyly of *R*. *schneideri *was recovered in 99.7% of the simulated mtDNA gene trees, and stands in contrast with the observed pattern of shared mtDNA sequences between this species and *R*. *marina *RAB populations. Accordingly, even after accounting for stochasticity and uncertainties in mutation rates (see Material and Methods), we can discard the hypothesis of incomplete lineage sorting as the explanation for the cytonuclear discordance reported here. Instead, we interpret our data as supporting an unidirectional massive mtDNA introgression between these species.

### Extensive unidirectional mtDNA introgression

Together with the current knowledge about the geographical distribution of both species, our results favour the hypothesis that the mitochondrial lineage detected in the right Amazon bank likely belonged to *R. schneideri *and was subsequently captured by *R. marina*. Notably, this hypothesis implicates that *R. schneideri *must have occupied a significant part of the Amazon and its extinction from this region was presumably preceded by a process of replacement driven by *R. marina *populations expanding southwards. A scenario supporting this hypothesis - under which such massive interspecific unidirectional mtDNA introgression could have taken place - has recently been proposed and modelled [32]. Following this model, when the territory of a resident species is invaded by another more successful species, even rare hybridization events at the front of this expansion can lead to introgression from the resident species into the expanding one. Drift at the front of the invasion and during the following expansion is responsible for repeated and transient secondary contacts among species, promoting situations of competitive replacement. In one of the most remarkable examples reported to date, Alves et al. [15] described how three Iberian hare species (*Lepus granatensis*, *L. europaeus *and *L. castroviejoi*) were extensively introgressed by the mtDNA of *L. timidus*, a boreal species presently extinct in Iberia. These authors indicated that *L*. *timidus *could have occurred in a significant part of Iberia during the LGM, but subsequently became extinct after climatic amelioration and competitively replaced by the more adapted temperate Iberian species that likely experienced a northwards range expansion [33].

The impact of Quaternary environmental changes in the Neotropical region has been the subject of extensive and controversial debate. In fact, there is a growing body of evidence originating in palaeoclimatological, palynological and palaecological data, but also in molecular phylogeographic studies [20], suggesting that significant changes in the Amazonian rainforest and Cerrado may have occurred during the late Pleistocene and the Holocene [34-36]. During previous drier glacial periods, the Cerrado expanded and may have penetrated into the Amazon forest, while most parts of the southern present-day Cerrado might have contracted due to strong cold fronts and to the expansion of subtropical grasslands [37,38]. Since the middle Holocene, a change towards more humid climatic conditions has occurred, promoting the expansion of the Amazon forest and also the southward re-establishment of Cerrado [39]. These climatic-induced changes seem to have played an important role on the evolutionary history of a great diversity of organisms [34,40,41]. For example, Ramos et al. [41] suggested that during glacial times the tree *Hymenaea stigonocarpa *became extinct in most parts of southern present-day Cerrado, being restricted to the milder climatic conditions of the northernmost and easternmost regions of this biome. After postglacial climate amelioration, this species expanded its range southwards. Accordingly, it is tempting to consider that recent climatic oscillations forced dramatic changes in species distributions, including the Neotropical toads *R. marina *and *R. schneideri*. These transient environmental conditions could have created the opportunity for these two species to meet and hybridize, a situation that may have been likely favoured by similar breeding behaviour and coincidence of reproductive seasons [42,43].

Following this hypothesis, the current distribution of *R. schneideri *(Figure 1A) seems to result from its recent retreat from the Amazon and a subsequent southward expansion into the present-day Cerrado biome, as evidenced by the low levels of mitochondrial diversity in all current distribution area. In fact, part of these populations is fixed for haplotype H14. Introgression from *R. schneideri *into *R. marina *could then correspond to phases of forest expansion, when the latter species would be favoured and eventually replaced the former [44]. The tendency toward negative values for Tajima's *D *and Fu's *F*s and low values for the R_2 _statistic observed in RAB populations across all loci could be interpreted as a signal of population expansion. In addition, indirect evidences that likely highlight the importance of climatic/ecological changes on the evolutionary history of these toad species are provided by some unique genetic signals present in Canaã do Carajás (CC). This population represents a small remnant island of the Cerrado biome within the humid Amazon forest [38,45] and correspond to the single location where we detected three individuals of *R. marina *having *R*. *schneideri RPL3 *haplotypes and the most frequent and widespread mtDNA haplotype of *R*. *schneideri *(H14). Taken together, this evidence may suggest that the population of Canaã dos Carajás could be located close to the putative hybrid zone between *R. marina *and *R*. *schneideri*. Future studies should clarify this question and, in particular, investigate if that hybrid zone is defined by a clear boundary between the dry and rain forest or corresponds instead to a more complex and patchy region due to the occurrence of multiple cerrado islands within the Amazonian forest.

### Implications of selective and neutral processes for asymmetric introgression

When introgression of mtDNA from one species into another occurs, foreign mtDNA can completely replace resident mtDNA through selective or neutral processes [4,46]. The effects of directional selection on mtDNA could produce changes in the patterns of genetic variability generating a star-like genealogy and a significantly skew towards low-frequency alleles [47-49]. Both Tajima's *D *and Fu's *Fs *tests of the frequency spectrum of mutations are significantly skewed towards rare alleles in the mitochondrial genealogy of *R. schneideri *(Table 1). There are many evidences that various sorts of selection pressures could act on mtDNA (reviewed in [50,51]), driving in some cases extensive mtDNA introgression [4]. However, this kind of deviation may be confounded by the influence of demographic history, which could have a similar impact on variability patterns under neutrality (e.g [4,14,33]). For example, deviations from a neutral model of DNA sequence variation closely linked to a site that has undergone a selective sweep may be similar to those in populations that experienced an expansion [50,52]. Both empirical and simulation studies have suggested that some alleles occurring at the front of a range expansion could travel with the wave of advance (surfing) over long distances and eventually reach high frequencies [33,53]. Likewise, a reduction in population size would also cause a depletion of genetic diversity by the action of genetic drift alone, which is more pronounced at mtDNA markers due to its lower effective size compared to nuclear markers (e.g [32,54]).

Other often reported explanations for differential introgression of mtDNA versus nuclear loci are male biased dispersal and dissortative mating, especially when prezygotic isolation models like female-preference or male-male competition are implicated [44]. However, such patterns could also be explained by postzygotic asymmetries (e.g [55]), in which reciprocal crosses resulted in different degrees of hybrid viability or fitness, or a combination of both pre- and postzygotic effects [56]. In amphibians, many examples of unidirectional introgression due to asymmetric reproductive behaviour have been reported, as expected in polygynous mating systems such as those found in bufonids ([57]; but see [58]). While to date natural hybridization in the *R. marina *group has only been demonstrated between *R. icterica *and *R. schneideri *[59], our data clearly suggest that this phenomenon is probably more widespread than reported so far. An extensive study involving more than 1900 laboratory crosses between 92 species of bufonids showed that crosses between *R. marina *females and both *R. poeppigii *and *R. arenarum *males resulted in hybrid offspring constituted only by females, while crosses involving *R. schneideri *females and *R. arenarum *males produced hybrids of both sexes [60,61]. While we cannot discard the possibility that asymmetric reproductive isolation could have resulted in the observed asymmetrical mtDNA introgression between *R. marina *and *R. schneideri*, further studies investigating the existence and the extent of pre- and/or postzygotic barriers between these species are required to examine this hypothesis.

## Conclusions

Our results clearly suggest that the closely related *R. marina *and *R. schneideri *shared a history of introgressive hybridization. Nuclear analyses support the monophyly of both species, a fact that would have not been perceived if our inferences were based solely on the mtDNA analysis. Another important inference of this work is that past environmental changes have likely played a crucial role for the occurrence of hybridization in the Neotropics. This is of special interest considering recent habitat changes due to extensive deforestation of the Amazonian forest in last decades for industrial and agriculture purposes. In fact, it has been demonstrated that human-induced environmental changes could create the opportunity to increase the area of sympatry and the likelihood of hybridization between many closely related taxa (e.g [62]). Future research focusing on transition areas between the Amazon rain forest and the dry forests of Cerrado and Caatinga will be crucial for a deeper understanding of the consequences of the complex past climate and present-day environmental changes on the evolutionary trajectory of organisms.

## Methods

### Taxon sampling and laboratory methods

A total of 95 tissue samples of *R. marina *(68) and *R. schneideri *(27) from different regions of Brazil and Paraguay were obtained through field work and also from museum preserved samples in the case of *R. schneideri *(Figure 1B; Additional file 1). Total DNA was extracted from each sample using the standard phenol-chloroform method, followed by sodium acetate precipitation [63]. We obtained sequences of the mitochondrial Cytochrome b (*cyt b*) gene, and the nuclear intron 6 of the ribosomal protein L9 (*RPL9int6*, thereafter *RPL9*), the intron 5 of the ribosomal protein L3 (*RPL3int5*, thereafter *RPL3*) and the partial exon and intron 2 of the cellular myelocytomatosis oncogene (*c-myc*). Sequences of the three nuclear genes were obtained for *Rhinella arenarum *and *Rhinella icterica*, which were used as outgroups [25]. The following primers were used for amplification and sequencing: for *cyt b *- primers CytbFor and CytbRev [64]; for *RPL9 *- primers RPL9intF and RPL9intR [12]; for *RPL3*-primers RPL5F and RPL36RA [12]; and for *c-myc *- primers Cmyc1U [65] and Cmyccat3 (5'- GTTGYTGCTGATCTGTTTGAG - 3') were used for initial amplification and sequencing. After this, internal primers were specifically designed for this study MarCmycF (5'-TGA TGC ATA GAC CCT TCG GTG-3') and MarCmycR (5'-GAT AGT CCG CTC TGG TGG AAG-3').

PCR conditions for *cyt b *amplification were done according to those reported in [25]. Amplifications were performed using ~10 ng of genomic DNA; Tris-HCl pH 8.85, 25 mM KCl; 5 mM MgCl_2_; 0.2 mM each dNTPs; 0.5 μM of each primer and 1 U Taq DNA polymerase (Invitrogen Corporation, Carlsbad, CA, USA). Amplification conditions of 94°C for 3 min, followed by 30 cycles of 94°C for 1 min, annealing temperature of 54°C for 1 min, 72°C for 1 min, and a single final step at 72°C for 5 min. The PCR conditions for *RPL9*, *RPL3 *and *c-myc *amplifications were the same of those described in [12]. PCRs were carried out in 10 μl volume containing 1× PCR buffer; 3 mM MgCl_2_; 0.4 mM each dNTPs; 0.5 U of GoTaq DNA polymerase (Promega); 0.3 μM each primer and approximately 50 ng of genomic DNA. Amplification conditions consisted of a pre-denaturing step of 5 min at 92°C followed by 40 cycles of a denaturing step of 30 s at 92°C, annealing temperature ranging between 50-54°C for 30 s and extension at 72°C for 90 s. The final extension was accomplished at 72°C for 5 min. PCR products were enzymatically purified with the ExoSap-IT purifying kit (Amersham-Pharmacia Biotech) and sequenced in both directions using BigDye 3.1 on an Applied Biosystems 3100 DNA automatic sequencer, according to protocols supplied by the manufacturers (Applied Biosystems, Foster, CA, USA).

### Sequence analysis

For nuclear sequences we used two approaches to resolve the haplotype phase: i) the method of [66] for sequences that were heterozygous for insertions or deletions; and ii) the Bayesian algorithm of PHASE [67] implemented in the DNAsp v.5 [68] using known phases of haplotypes determined by the previous method. This analysis was run multiple times (3) with different seeds for the random-number generator and checked if haplotype estimation was consistent across runs. Each run was conducted for 1.0 × 10^6 ^iterations with the default values. Haplotypes with a probability less than 0.95 probabilities were excluded from the analysis. For all the analyses we completely removed indels because they did not significantly reduce the number of polymorphic sites, nor disregard information contained in the data sets. After these procedures, sequences were edited and aligned in BioEdit v. 5.0.6 [69]. Alignments of protein coding gene (*cyt b*) were unambiguous with no insertions or deletions. All the sequences obtained in this study were deposited in GenBank under numbers JN594508-JN594607.

For both mtDNA and nuclear fragments we calculated summary statistics in DNAsp, which include: *π*, nucleotide diversity [70]; *θ*, Watterson population mutation parameter [71]; S, number of segregating sites, and Fu's *Fs *[47], Tajima's *D *[49] and R_2 _[48] neutrality tests. Significance values of all statistical tests were computed using the coalescent simulator implemented in DNAsp by comparing estimated values against a distribution generated from 10,000 random samples under the hypothesis of selective neutrality and population equilibrium, with no recombination [72]. To evaluate the possibility of recombination we computed the Rm statistic (minimum number of recombination events) [73] using DNAsp. We also used the software PHIPACK to test for recombination using the pairwise homoplasy index (*Φw *statistic) [74], because Rm is likely to be highly affected by homoplasy. We used the permutation test (1000 permutations) to estimate P-values.

Genealogical relationships among haplotypes for each *locus *were first estimated using a network-approach. Haplotype networks were estimated using the median joining (MJ) method [75] as implemented in the software NETWORK v. 4.1.1.2 [76]. However, because networks applying the aforementioned method recovered many unresolved loops in the genealogical connections between haplotypes (Additional file 3), we generated haplotype networks using phylogenetic algorithms with migration and using proper models of sequence evolution [77]. Phylogenetic reconstructions among haplotypes for each locus were estimated using a Maximum Likelihood (ML) approach, as implemented in the software PHYML 3.0 [78]. Using default options we ran the program with the best fit model for each locus as selected by Kakusan4 [79]. The generated trees were used to estimate each network haplotype in Haploviewer program [77].

### Divergence time estimates and coalescent simulations

The use of Isolation-with-Migration model (IMa2) involving more than two populations requires a rooted phylogenetic tree with known sequence in time of internal nodes [80]. Accordingly, we first reconstructed a phylogenetic tree based on the three nuclear genes using the species tree inference methodology *BEAST [81], as implemented in BEAST v1.6.1 [82]. In *BEAST, the assignment of specimens to taxa must be given as a prior for the analysis. For this purpose, we defined three groups: i) *R. marina *sequences were divided in the two different geographic groups of populations, RAB and LAB; and, ii) all *R*. schneideri sequences were defined as a group, The toads *R. icterica *and *R. arenarum *were used as outgroups [24,25]. The input file for *BEAST was created using the application BEAUti [82] and partitions and models were edited by hand to fit models for each unlinked nuclear fragment, as determined with Kakusan4. Posterior phylogenies were determined in *BEAST using a relaxed lognormal clock model and the prior was set to the default option of Yule process [83]. All remaining priors were set to the defaults. Three replicate runs of 500 million generations were conducted, sampling trees and parameter estimates every 50,000 generations. Convergence was checked using Tracer v1.5 [84] and summary trees were generated with TreeAnnotator v1.6.1 [82].

We used IMa2 [80] to estimate the divergence time (t) between: i) *R. marina *and *R*. *schneideri *[RM (LAB + RAB) × RS] using combined information from the three nuclear loci; ii) populations of *R. marina *from left (LAB) and right (RAB) Amazon bank LAB × RAB using information from all three nuclear loci; iii) populations of *R. marina *from left (LAB) and right (RAB) Amazon bank + *R. schneideri *[LAB × (RAB + RS)] using mtDNA alone; and iv) *R. marina *and *R. schneideri *combining all markers and assuming that mtDNA from RAB population belong to *R. schneideri *[RM (LAB + RAB) × RS (RAB + RS)] (see Table 3). We used the HKY model of evolution [85], which takes into account the possibility of multiple hits, differences in nucleotide frequencies and the presence of transition/transversion bias. We ran the program under Metropolis Coupled MCMC, using ten chains with linear heating mode. Multiple preliminary runs were conducted to assess mixing of the chains, as well as to determine appropriate priors for the parameters. After this, we ran the program multiple times with different random seeds and for each simulation the length was > 10 × 10^6 ^steps, where the first 10 × 10^5 ^was discarded as burn-in. Convergence by the Markov chain simulations to stationary distribution was checked by monitoring multiple independent runs for each data set using different random number seeds (similar posterior distributions for each parameter across independent runs) and by assessing effective sample sizes (ESSs) values (ESS > 100), trendline plots, and swapping rates between chains over the course of the run. The estimates for *t *were converted into time in years since divergence (t) using the equation, t = t*μ, where μ is the neutral mutation rate for the locus.

Inferred rates of sequence divergence for *cyt b *of amphibians were estimated by Lougheed et al. [86], and range from 0.8% to 2.5% per Myr. In particular, Bufonid mtDNA evolution has been estimated [87] at about 1.38% sequence divergence based on divergence for the ND1+tRNA's mitochondrial region between *Bufo gargarizans *(from the eastern Tibetan Plateau) and *Bufo viridis *(an European species) and the estimated time for the vicariant event caused by the uplifting of the Tibetan Plateau [87]. Although these molecular evolutionary rates were estimated using different genes than the ones used here [88], comparing the sequence divergence between those taxa for 519 bp of *cyt b *[89] showed that the rates among these two genes are roughly comparable. Therefore, we considered reasonable to assume a substitution rate of 1.38% per Myr (0.69 × 10^-8 ^site/Myr) in this study. Similarly, substitution rates for all nuclear markers used here are unknown for *Rhinella *species group. Nevertheless, the rate of evolution for the same *c-myc *region used in this study has been estimated to be 2.01 × 10^-9 ^site/Myr based on sequence divergence between the Central and South American species of the Neotropical frog *Eleutherodactylus *[90]. Although nuclear mutation rates are quite variable among genes and organisms, a value of ≈ 2 × 10^-9 ^substitutions/site/year has been estimated for a wide range of vertebrates [91-93]. Thus, in this study we choose the mutation rate estimates reported for the Neotropical amphibians [90]. Because we only have calibrations for *c-myc *among the nuclear dataset and for mitochondrial *cyt b *gene, we used mutation rate scalars estimated in IMa2. For conversions, we multiplied t by the mutation rate scalars for *c-myc *and *cyt b *and then divided this value by the geometric mean of the mutation rates for the two loci.

Although temporal and demographic estimates using the above described approach have been widely applied, we must acknowledge that scalar manipulations of estimated mutation rates could be a potential source of inference error. In fact, accurate estimation of divergence times is especially critical in the absence of well-defined calibration points from independent data (e.g., dated fossil records, known biogeographic events, or paleoclimatic reconstructions), or due to the stochastic nature of molecular substitution rates. Despite this, we consider this approach as a valuable tool to provide a more comprehensive view of the temporal window and historical demography of our species.

In order to assess whether the phylogenetic conflicting signal found between mtDNA and nuclear DNA could be explained by incomplete lineage sorting, we tested the possibility of recovering the phylogenetic pattern inferred from mtDNA under the speciation scenario inferred from nuclear DNA. For that, we simulated mtDNA sequence datasets under a model with no gene flow and the population history (species-tree) and parameter estimates (IMa2) inferred from nuclear DNA, using SimCoal2 [94]. Estimates of current and ancestral effective population sizes Ne) and divergence times (t) obtained under an Isolation-with-Migration model (IMa2) were used as input for SimCoal2. A total of 10,000 simulated mtDNA sequence datasets, mimicking the empirical mtDNA datasets (sequence lengths and sample sizes), under a model with two historical events were produced: i) an ancestral haploid population of effective size N_eA_/2 split into N_e1_/2 (LAB) and N_e2_/2 (RAB) populations at t generations ago and, ii) an ancestral haploid population of size N_eA_/2 split into two descendants of size N_e1_/2 (*R. marina*; RAB+LAB) and N_e2_/2 (*R. schneideri*), t generations ago. Since we did not obtain accurate estimates for N_eA _of *R*. *marina *and *R. schneideri*, we considered N_eA _as the average between *R. marina *and *R*. *schneideri *effective population sizes. We used the mtDNA mutation rate (μ) aforementioned and a generation time of one year [43]. Theta (θ) was converted to Ne using the equation θ = 4Neμ. Sequences were generated using a mutation model with unequal transition-transversion rate (the transition proportion was determined for the empirical datasets with Kakusan4. Garli v1.0 [95] was used to reconstruct mtDNA Maximum Likelihood trees from the data simulated in each of the simulated data replicates. Garli was set to run until no significantly better scoring topology (as defined by the default settings) was encountered after 5,000,000 generations. Five replicate runs were performed to check for the consistency of the estimates. We rejected the incomplete lineage sorting scenario if the non-monophyly of *R. schneideri *(the pattern observed with the empirical mtDNA dataset) was inferred in less than 5% of the simulated datasets.

## Authors' contributions

FS, MV, DS carried out molecular laboratory work. JARB and DS collected samples. FS, MV and NF analysed the data, and drafted the manuscript. All authors participated in the conception and design of the study, writing and approval of the final manuscript.

## Supplementary Material

Additional file 1 **Sample size (N) and haplotypes (H) found in each sampled populations for all sequenced loci**. Number between parentheses represents the number of individuals sharing the same haplotype. Population code is represented as in Figure 1. Click here for file

Additional file 2 **The posterior probability distributions of divergence time (t), theta (*θ*) and population migration rates (2Nm) estimates using Isolation-with-Migration model (IMa2) for five pairwise comparisons**. (A) *R. marina *and *R. schneideri *[RM (LAB + RAB) × RS], and *R. marina *from left (LAB) and right (RAB) Amazon bank (LAB × RAB) using nuclear loci. Posterior probability of 2Nm from LAB to RAB is represented in the right vertical axis; (B) *R. marina *from left (LAB) and right (RAB) Amazon bank (LAB × RAB) using mtDNA *cyt b *gene; (C) *R. marina *from left (LAB) and right (RAB) Amazon bank + *R*. *schneideri *[LAB × (RAB + RS)] using mtDNA data; (D) *R. marina *and *R. schneideri *combining all markers and assuming that mtDNA from RAB belongs to *R. schneideri *[RM (LAB + RAB) × RS (RAB + RS)]. Click here for file

Additional file 3 **Median-joining haplotype networks**. (A) *cyt b*; (B) *RPL9*; (C) *RPL3; *and, (D) *c-myc*. The circle area of each haplotype, coded as a number (Additional file 1), is proportional to its frequency. White and black haplotypes are present in *R. marina *from Left Amazon river bank (LAB) and Right Amazon river bank (RAB), respectively, and gray haplotypes are present in *R. schneideri*. Median vectors are represented by red dots. Black dots represent inferred unsampled or extinct haplotypes. Click here for file
