# Supplementary material for: Hybridization and massive mtDNA unidirectional introgression between the closely related Neotropical toads Rhinella marina and R. schneideri inferred from mtDNA and nuclear markers
Source: BMC Evol Biol. 2011 Sep 22;11:264. doi: 10.1186/1471-2148-11-264 (PMC3192708; doi:10.1186/1471-2148-11-264)
Supplement: Additional file 1 — Sample size (N) and haplotypes (H) found in each sampled populations for all sequenced loci. Number between parentheses represents the number of individuals sharing the same haplotype. Population code is represented as in Figure 1. [file 1471-2148-11-264-S1.PDF]

Additional file 1. Sample size (N) and haplotypes (H) found in each sampled populations for all sequenced loci. Number between parentheses represents the number of individuals sharing the same haplotype. Population code is represented as in Fig. 1.

Populations	*N*	*Cyt b*	*C-myc*	*RPL3*	*RPL9*
Rhinella marina					
PT- Porto Trombetas, Pará	16	H1 (3)<br>H2 (6)<br>H3 (5)<br>H4 (1)<br>H5 (1)	H1 (1)<br>H2 (4)<br>H3 (1)	H1 (2)<br>H2 (3)<br>H3 (1)<br>H4 (1)<br>H5 (3)	H1 (2)<br>H2 (2)<br>H3 (1)<br>H4 (3)<br>H5 (1)<br>H6 (1)
BA – Bailique, Amapá	6	H1(6)	H1 (2)<br>H2 (2)	H6 (6)	H2 (9)<br>H7 (1)
ST - Santarém, Pará	10	H6 (9)<br>H7 (1)	H2 (10)<br>H4 (2)	H4 (4)<br>H9 (2)<br>H15 (1)<br>H16 (1)	H1 (1)<br>H2 (3)<br>H8 (2)<br>H9 (2)<br>H10 (2)
CC- Canaã dos Carajás, Pará	10	H9 (4)<br>H12 (1)<br>H13 (2)<br>H14 (3)	H2 (5)<br>H10 (3)	H7 (5)<br>H8 (2)<br>H9 (2)<br>H10 (5)<br>H11 (2)<br>H12 (1)<br>H13 (1)<br>H14 (2)	H2 (2)<br>H6 (1)<br>H12 (4)<br>H17 (1)<br>H18 (3)<br>H19 (1)
SO – Soure, Pará	4	H9 (2)<br>H10 (1)	H1 (2)<br>H2 (2)<br>H3 (2)<br>H9 (2)	H7 (4)<br>H8 (1)<br>H10 (5)<br>H17 (1)<br>H19 (1)	H10 (3)<br>H14 (3)
AL – Algodoal, Pará	10	H8 (5)<br>H9 (5)	H2 (3)<br>H3 (1)	H7 (3)<br>H8 (1)<br>H10 (3)<br>H17 (1)<br>H18 (2)	H2 (1)<br>H14 (2)<br>H15 (1)<br>H16 (1)<br>H17 (1)
VI – Viseu, Pará	10	H9 (5)<br>H10 (3)<br>H11 (2)	H2(4)<br>H5 (2)<br>H6 (2)<br>H7 (1)<br>H8 (1)	H7 (4)<br>H8 (2)<br>H10 (8)	H8 (5)<br>H11 (1)<br>H12 (2)<br>H13 (1)<br>H14 (1)

Rhinella schneideri					
AR – Araguaçema, Tocantins	3	H14 (2)<br>H15 (1)	-	H25 (2)	H20 (2)<br>H24 (2)
PO – Pontalina, Goiás	2	H14 (2)	-	H21 (2)<br>H25 (2)	H20 (1)<br>H24 (1)
MN - Mundo Novo, Mato Grosso do Sul	12	H14 (12)	H11 (5)<br>H12 (5)<br>H13 (1)	H20 (2)<br>H21 (2)<br>H22 (3)<br>H23 (3)<br>H24 (2)	H20 (5)<br>H21 (1)<br>H22 (1)<br>H23 (1)
RC - Rio Claro, São Paulo	4	H14 (4)	H11 (3)	H22 (2)<br>H25 (1)<br>H26 (1)	H20 (8)
AM – Amambay, Paraguay	3	H16 (3)	H14 (4)<br>H15 (1)<br>H16 (1)	H21 (1)<br>H24 (1)<br>H27 (3)<br>H28 (1)	H25 (1)<br>H26 (1)<br>H27 (1)<br>H28 (1)
LI – Limoy, Paraguay	2	H17 (1)	H11 (1)<br>H13 (1)<br>H14 (2)	-	H24 (1)<br>H29 (1)
IT- Itapuá, Paraguay	1	H6 (1)	-	H23 (1)<br>H28 (1)	H27 (1)<br>H30 (1)
**Outgroups**					
Rhinella arenarum *<br>(Rocha, Uruguay)		GU178807	*Rar2*	*Rar2*	*Rar2*
Rhinella icterica *<br>(S. Paulo, Brazil)		GU178808	*Ric1*	*Ric1*	*Ric1*

* GenBank accession number and sample code [25].
