# Supplementary material for: Hybridization and massive mtDNA unidirectional introgression between the closely related Neotropical toads Rhinella marina and R. schneideri inferred from mtDNA and nuclear markers
Source: BMC Evol Biol. 2011 Sep 22;11:264. doi: 10.1186/1471-2148-11-264 (PMC3192708; doi:10.1186/1471-2148-11-264)

Additional file 2A

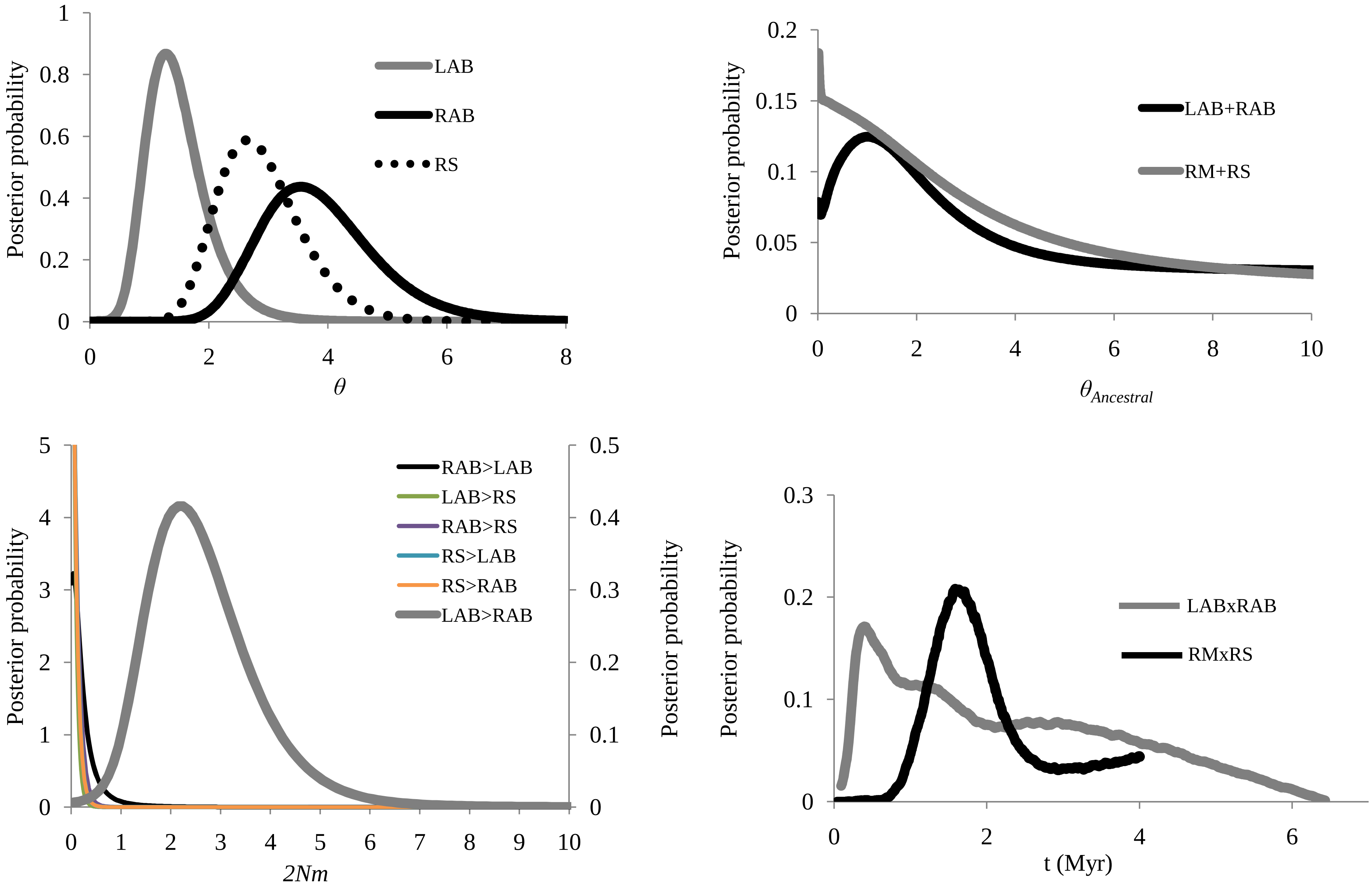

Additional file 2B

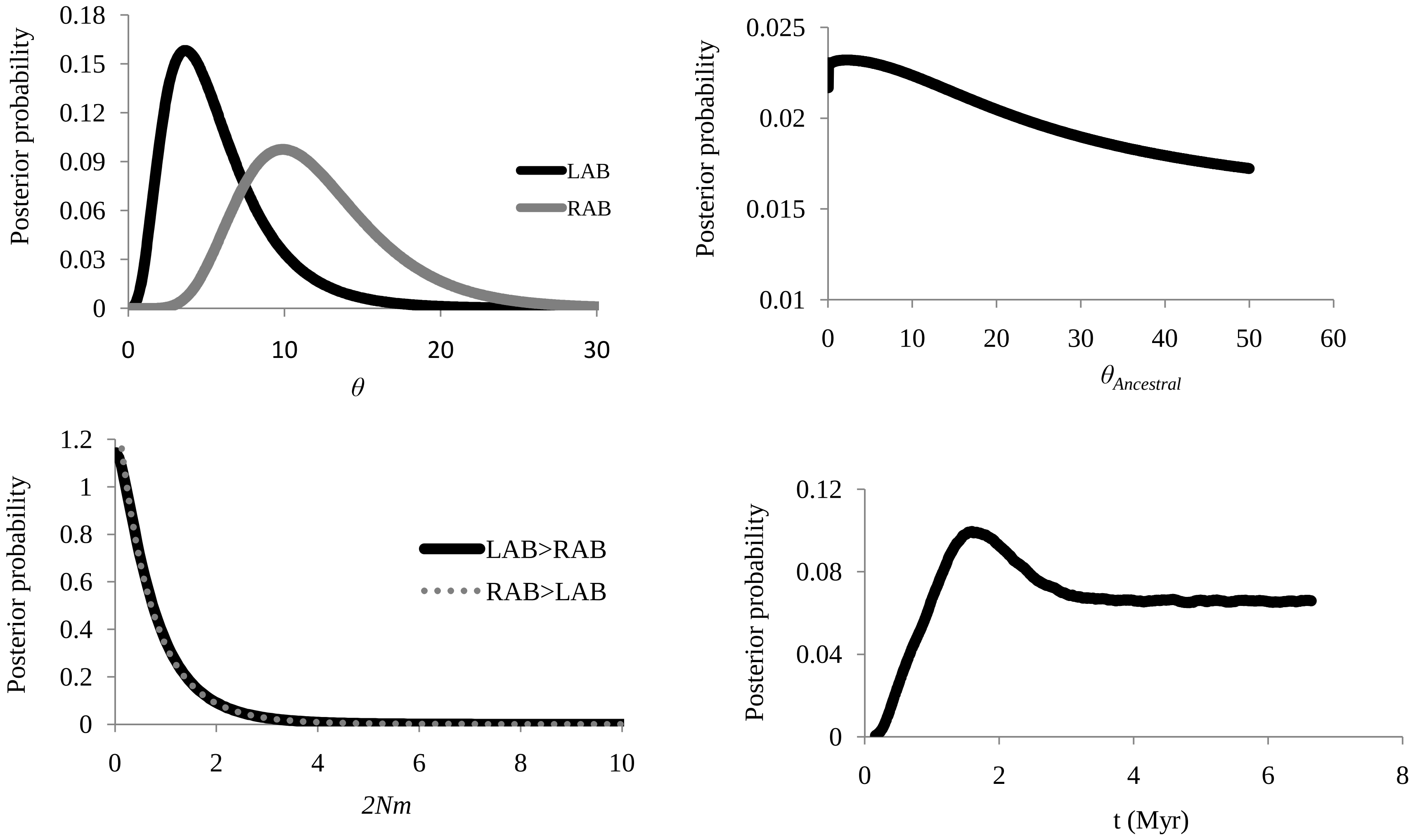

Additional file 2C

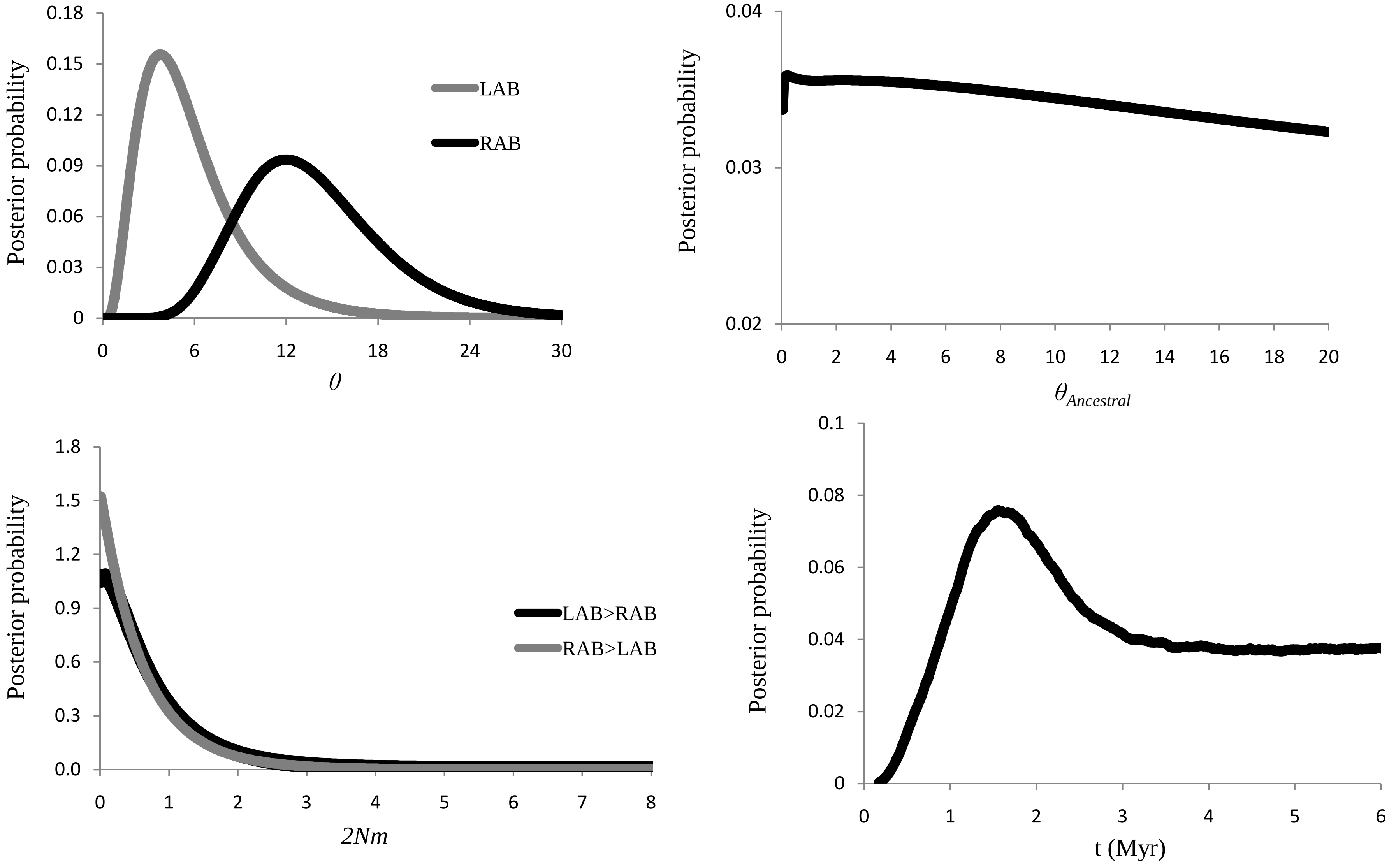

Additional file 2D

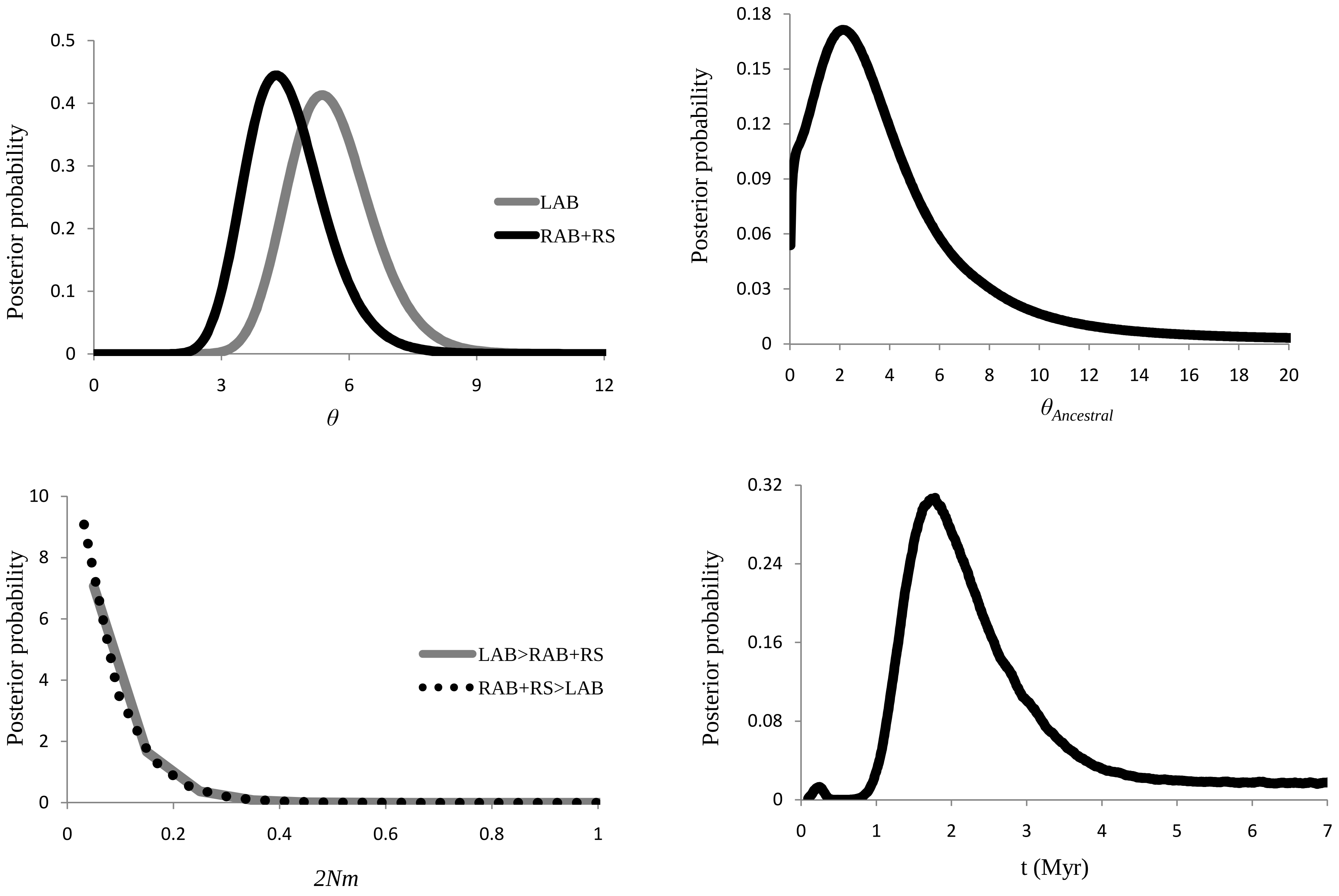

Supplement: Additional file 2 — The posterior probability distributions of divergence time (t), theta (θ) and population migration rates (2Nm) estimates using Isolation-with-Migration model (IMa2) for five pairwise comparisons. (A) R. marina and R. schneideri [RM (LAB + RAB) × RS], and R. marina from left (LAB) and right (RAB) Amazon bank (LAB × RAB) using nuclear loci. Posterior probability of 2Nm from LAB to RAB is represented in the right vertical axis; (B) R. marina from left (LAB) and right (RAB) Amazon bank (LAB × RAB) using mtDNA cyt b gene; (C) R. marina from left (LAB) and right (RAB) Amazon bank + R. schneideri [LAB × (RAB + RS)] using mtDNA data; (D) R. marina and R. schneideri combining all markers and assuming that mtDNA from RAB belongs to R. schneideri [RM (LAB + RAB) × RS (RAB + RS)]. [file 1471-2148-11-264-S2.PDF]
