# Supplementary figures and images for: Hybridization and massive mtDNA unidirectional introgression between the closely related Neotropical toads Rhinella marina and R. schneideri inferred from mtDNA and nuclear markers
Source: BMC Evol Biol. 2011 Sep 22;11:264. doi: 10.1186/1471-2148-11-264 (PMC3192708; doi:10.1186/1471-2148-11-264)

## Additional file 3

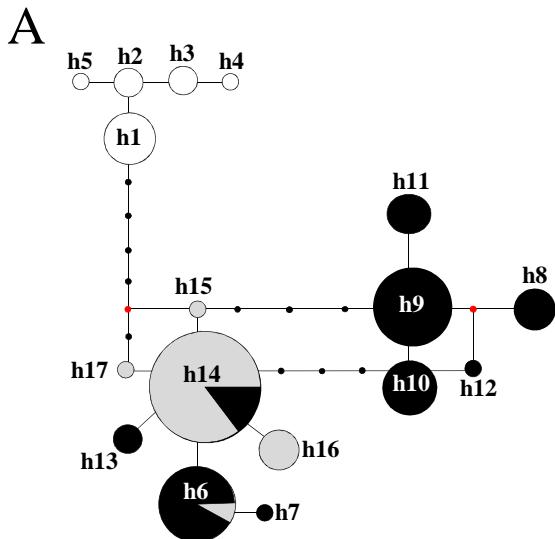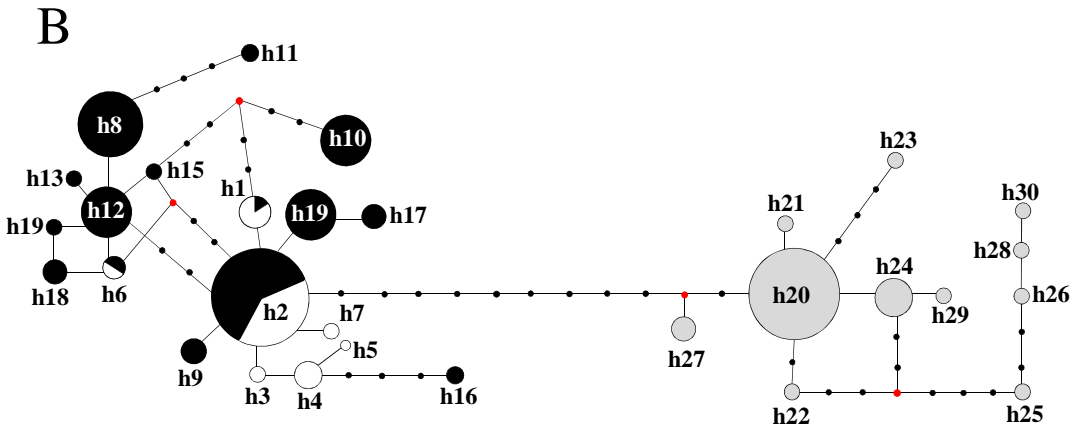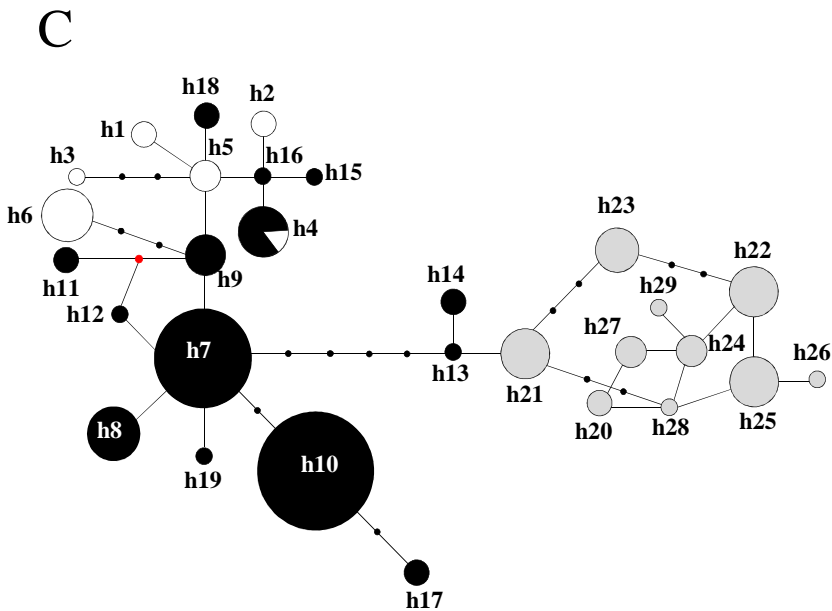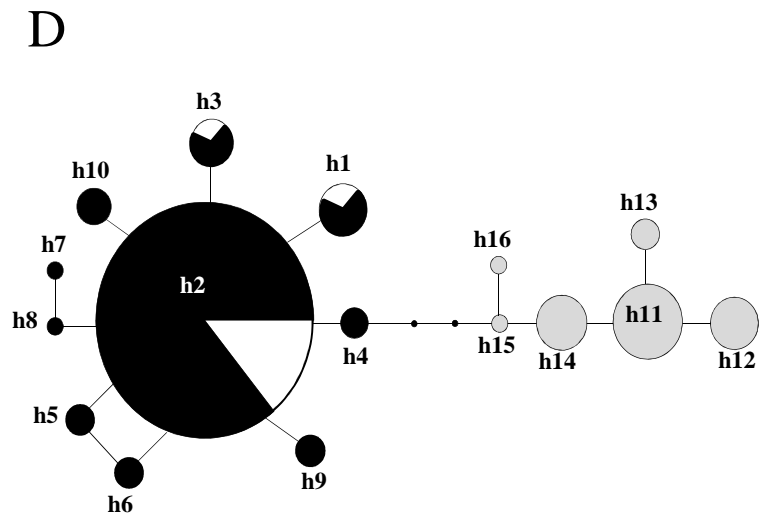

Supplement: Additional file 3 — Median-joining haplotype networks. (A) cyt b; (B) RPL9; (C) RPL3; and, (D) c-myc. The circle area of each haplotype, coded as a number (Additional file 1), is proportional to its frequency. White and black haplotypes are present in R. marina from Left Amazon river bank (LAB) and Right Amazon river bank (RAB), respectively, and gray haplotypes are present in R. schneideri. Median vectors are represented by red dots. Black dots represent inferred unsampled or extinct haplotypes. [file 1471-2148-11-264-S3.PDF]
